# Pharmacokinetics and Excretion of Berberine and Its Nine Metabolites in Rats

**DOI:** 10.3389/fphar.2020.594852

**Published:** 2021-01-15

**Authors:** Xinchi Feng, Kun Wang, Shijie Cao, Liqin Ding, Feng Qiu

**Affiliations:** ^1^ School of Chinese Materia Medica, Tianjin University of Traditional Chinese Medicine, Tianjin, China; ^2^ Tianjin State Key Laboratory of Modern Chinese Medicine, Tianjin University of Traditional Chinese Medicine, Tianjin, China

**Keywords:** berberine, metabolites, pharmacokinetics, excretion, rats

## Abstract

Berberine, a well-known alkaloid, has been proved to possess various pharmacological activities. Previous studies demonstrated that berberine could be extensively metabolized and the metabolites also contributed to its therapeutic effects. However, as for berberine’s metabolites, especially phase II metabolites, pharmacokinetics and excretion studies were rarely reported. The objective of this study was to thoroughly investigate the pharmacokinetic and excretion profiles of berberine and its nine metabolites, namely, berberrubine (M1), demethyleneberberine (M2), jatrorrhizine (M3), jatrorrhizine-3-*O-β*-D-glucuronide (M4), jatrorrhizine-3-*O*-sulfate (M5), thalfendine-10-*O-β*-D-glucuronide (M6), berberrubine-9-*O-β*-D-glucuronide (M7), demethyleneberberine-2-*O*-sulfate (M8) and demethyleneberberine-2-*O-β*-D-glucuronide (M9) in rats. An accurate and reliable LC-MS/MS method was developed and validated for the determination of berberine and its nine metabolites in rat biosamples. Pharmacokinetic profiles of berberine and its nine metabolites were obtained after a single intravenous administration (4.0 mg/kg) and oral administration (48.2, 120 or 240 mg/kg) of berberine in rats. For excretion study, rats were intragastrically administered a single dose of 48.2 mg/kg berberine. Our results showed that berberine could be metabolized rapidly and all the nine metabolites could be detected *in vivo*. The absolute bioavailability of berberine was 0.37 ± 0.11%. As for the AUC_0–48 h_ values, phase II metabolites were much higher than those of phase I metabolites, suggesting that phase II metabolites were the major metabolites exist in blood circulation. 18.6% of the berberine was excreted in feces as berberrubine (M1). The total recovery of berberine and its nine metabolites from urine, bile and feces was 41.2%. This is the first systematic study about the pharmacokinetics and excretion of berberine and its nine metabolites, which will be beneficial for both better understanding the clinical effects and further development of berberine.

## Introduction

**FIGURE 1:**
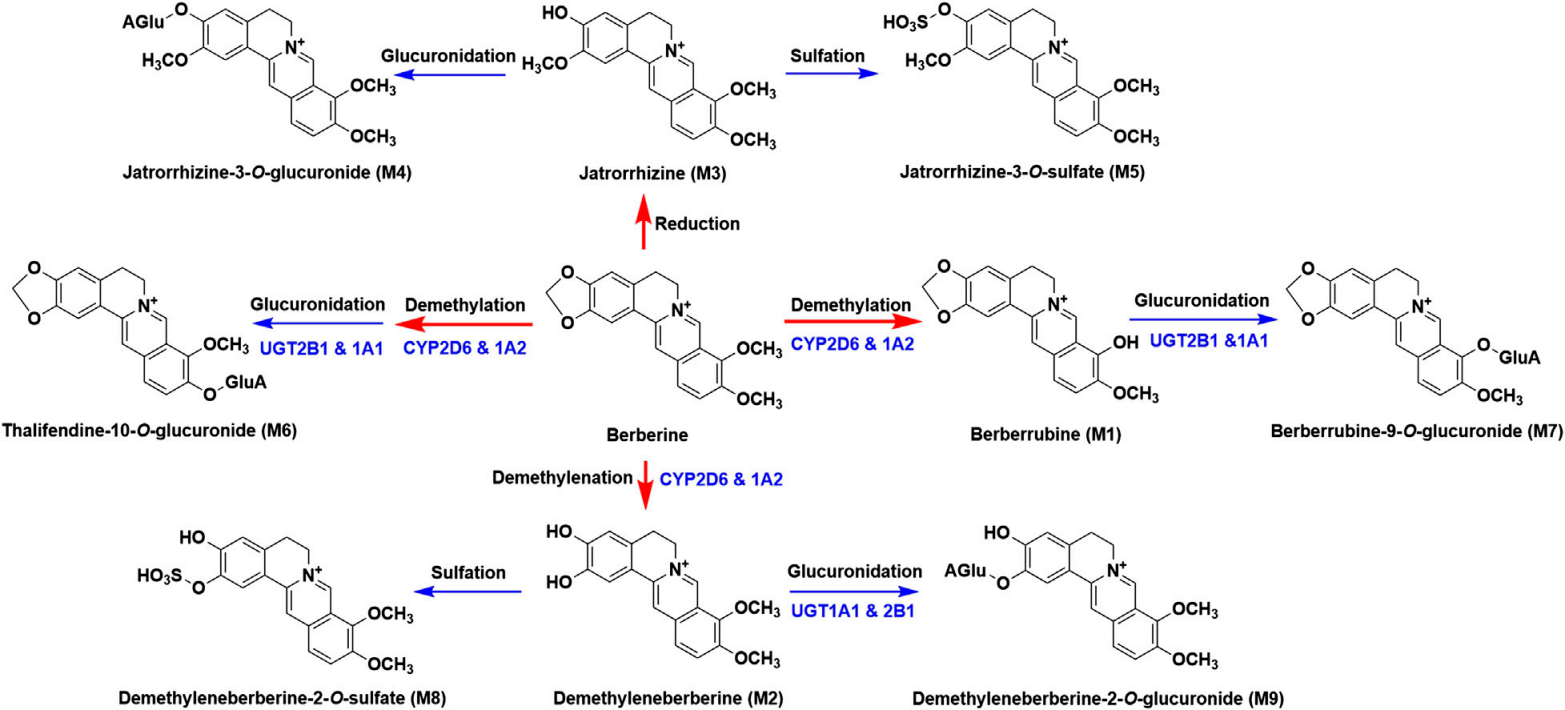
Chemical structures of berberine and its nine metabolites (M1 to M9).

Berberine (Figure 1), a well-known alkaloid, is the principle component found in various medicinal herbs such as *Coptis chinensis* Franch. (Chen et al., 2008), *Coptis japonica* (Thunb.) Makino (Min et al., 2006), and *Phellodendron chinense* C. K. Schneid (Liu Y. et al., 2010). It has a long history of medicinal use in traditional Chinese medicine for its remarkable efficacy, including clearing heat, purging fire, drying dampness, cooling blood, and removing toxicity. Modern pharmacological studies have revealed that berberine also possesses anti-diabetic (Yin et al., 2008), hypolipidemic (Kong et al., 2004), anti-inflammatory (Habtemariam, 2016), immunosuppressive (Li et al., 2016) and anti-cancer (Liu et al., 2019) activities. In addition to Asian countries, berberine has currently received great interest in European (Och et al., 2020) and also gained entry in African countries as a dietary supplement (Alolga et al., 2016).

Pharmacokinetic studies of berberine have been conducted by several investigators, and it has been revealed that berberine undergoes extensive metabolism and the oral bioavailability of berberine is really low (Zuo et al., 2006; Hua et al., 2007; Liu et al., 2010; Ma et al., 2013; Tan et al., 2013; Feng et al., 2018). The contradiction between the definite curative effect of berberine and its the extremely low plasma concentration promoted the hypothesis that the metabolites of berberine may also contribute to its bioactivities. This hypothesis had attracted a wide range of attention from numerous researchers and been proved by our previous studies and other investigators’ studies (Cao et al., 2013; Zhou et al., 2014; Yang et al., 2017). As for the phase I metabolites of berberine, demethyleneberine was proved to show anti-inflammatory and hepatoprotective effects and could be serve as a potential therapy for liver disease, hepatic fibrosis and autoimmune hepatitis (Zhang et al., 2015; Qiang et al., 2016; Wang et al., 2016; Chen et al., 2017; Zhang et al., 2020). Berberrubine was reported to show lipid-lowering and anti-ulcerative colitis effects (Cao et al., 2018; Yu et al., 2018). Jatrorrhizine, columbamine and palmatine were the main active components exist in many herbs had been proved to exert similar bioactivities of berberine (Wang et al., 2017b). As for the phase II metabolites of berberine, berberrubine-9-*O-β*-D-glucuronide showed excellent glucose-lowering effect (Yang et al., 2017). Thus, comprehensive characterization of the pharmacokinetic and excretion profiles of berberine and its metabolites is very essential for its clinical use.

According to our previous study, berberine could be metabolized into 97 metabolites in rats (Wang et al., 2017a). Even though the pharmacokinetics of berberine has been extensively investigated, the pharmacokinetics of berberine’s metabolites was rarely reported. The limited studies available mainly focused on the unchanged berberine and its phase I metabolites, namely, berberrubine, demethyleneberberine, jatrorrhizine, thalifendine and palmatine (Zuo et al., 2006; Ma et al., 2013; Feng et al., 2018). Until now, the pharmacokinetic profiles of phase II metabolites of berberine have not been revealed. However, our preliminary study showed that compared with the phase I metabolites, the plasma concentrations of phase II metabolites were much higher, which was consistent with the reported results (Zuo et al., 2006). Additionally, the phase II metabolites of berberine have been proved to possess pharmacological activities. For example, berberrubine-9-*O-β*-D-glucuronide showed glucose-lowering effect and it was suggested that its glucose-lowering effect was much greater than that of berberrubine (Yang et al., 2017). Hence, characterization the pharmacokinetic profiles of berberine’s metabolites, especially phase II metabolites, will be helpful to better understand its clinical efficacy. In our study, pharmacokinetics and excretion of berberine and its nine major metabolites (three phase I and six phase II metabolites, Figure 1), namely, berberrubine (M1), demethyleneberberine (M2), jatrorrhizine (M3), jatrorrhizine-3-*O-β-*D-glucuronide (M4), jatrorrhizine-3-*O*-sulfate (M5), thalfendine-10-*O-β*-D-glucuronide (M6), berberrubine-9-*O-β*-D-glucuronide (M7), demethyleneberberine-2-*O*-sulfate (M8) and demethyleneberberine-2-*O-β*-D-glucuronide (M9) were carried out in rats. This study will help to better understand the *in vivo* disposition of berberine.

## Materials and Methods

### Chemicals and Reagents

Berberine sulfate and berberine chloride (99.5% purity) were purchased from the Northeast General Pharmaceutical Factory (Shenyang, China). Jatrorrhizine chloride (98% purity) were obtained from Vientiane Tianjin Hengyuan Technology Co., Ltd. (Tianjin, China). Berberubine and demethyleneberberine (98% purity) were obtained from Shanghai Yuanye Bio-Technology Co., Ltd. (Shanghai, China). Tetrahydropalmatine purchased from Tianjin Shilan Technology Co., Ltd. was used as internal standard (IS). Methanol and acetonitrile (Fisher Scientific, United States) were of HPLC grade. Ammonium acetate and formic acid were purchased from Sigma-Aldrich Co. Ltd. (Poole, United Kingdom). Ultrapure water (Watsons, Guangzhou, China) was used throughout the study. Demethyleneberberine-2-*O*-sulfate, demethyleneberberine-2-*O-β*-D-glucuronide, berberrubine-9-*O-β*-D-glucuronide, thalifendine-10-*O-β*-D-glucuronide, jatrorrhizine-3-*O-β*-D-glucuronide, and jatrorrhizine-3-*O*-sulfate (purities were >95% by HPLC analyses) were isolated and prepared in our laboratory (Qiu et al., 2008). Other chemicals were of analytical grade.

### Animals, Drug Administration and Sampling

Sprague-Dawley rats (male, weighted 220–250 g) were obtained from the Beijing Military Medical Science Academy. All animals were maintained under controlled conditions (22 ± 2 °C, 45% relative humidity and a 12 h light/dark cycle) and fasted (free access to water) for at least 12 h prior to drug administration. Animal experiments were reviewed and approved by the Animal Research Ethics Committee of the Tianjin University of Traditional Chinese Medicine prior to the study.

For pharmacokinetic study, twenty-four rats were divided into four groups (*n* = 6). Berberine chloride (suspended in 0.5% CMC-Na) was administered intragastrically at a single dose of 48.2, 120, and 240 mg/kg to groups one to three, respectively. The fourth group was intravenously administered with a single dose of berberine sulfate (dissolved in saline solution of 0.9% NaCl) at 4.0 mg/kg. The selection of the dosages was based on the clinical dosages of berberine used in humans (Imenshahidi and Hossseinzadeh, 2019). In fact, various dosages of berberine were used for the treatment of different diseases in humans. The most commonly used dosage was 500 mg and the maximum dosage was 1.2 g for human. Thus, according to the body surface area scaling, 48.2 and 120 mg/kg (approximately equal to 500 mg and 1.2 g for human) were selected as the low and medium dosages for rats in this study. Meanwhile, 240 mg/kg (twice the medium dose) was chosen as the high dose in this study. Before the collection of blood samples, rats were anesthetized with isoflurane for about 30 s. Blood samples (around 300 μL) were collected from the eye venous plexus into tubes containing EDTA (about 5 mg in each tube) at 0, 0.25, 0.5, 1, 1.5, 2, 3, 4, 6, 8, 12, 24, 36, and 48 h after oral administration and at 0.083, 0.25, 0.5, 0.75, 1, 1.5, 2, 3, 4, 6, 8, 12, 24, 36, and 48 h following intravenous administration. Plasma was obtained by centrifugation at 4,000 rpm for 10 min and stored at −80 °C until analysis.

For excretion study, six rats were administered orally with a single dose of berberine chloride (suspended in 0.5% CMC-Na) at 48.2 mg/kg. After dosing, rats were housed in individual metabolism cages which allowed the separate collection of urine and feces. Urine samples were collected during intervals of 0–4, 4–8, 8–12, 12–24, 24–36, 36–48, 48–60, 60–72, and 72–84 h post-dosing and fecal samples were collected at 0–84 h post-dosing. After the collection of urine and fecal samples, the rats were allowed a washout period of 7 days and then employed in the biliary excretion study. After the rats were anesthetized with 20% urethane (*w/v*, *i.p.*), an abdominal incision was made and the bile duct was cannulated with tube to collect bile. A single 48.2 mg/kg dose of berberine chloride was orally administered to rats and bile samples were collected during intervals of 0–2, 2–4, 4–8, 8–12, 12–24, and 24–36 h post-dose. Samples were stored at −80 °C refrigerator until analysis.

### Sample Processing

For plasma, urine and bile samples, similar sample preparation procedure was employed. An aliquot of 50 μL each sample was spiked with 200 μL of precipitant containing 5.00 ng/mL of IS. After vortexed for 2 min, the mixture was centrifuged at 14,000 rpm for 10 min. 150 μL of supernatant was transferred to a new tube and diluted with 50 μL (for plasma sample) or 100 μL (for urine and bile sample) of distilled water. Fecal samples were freeze-dried, weighted, pulverized into powder and ultrasonically extracted with methanol (1:10, *w/v*) for 30 min. Then, fecal sample extracts were centrifuged at 3,000 rpm for 10 min and an aliquot of 50 μL supernatant was transferred to a new tube and treated in the same way as the urine samples. All samples were vortexed for 2 min before LC-MS/MS analysis and the sample injection volume was 2 μL. For the determination of berberine, M1, M2, and M3, acetonitrile was used as precipitant, and for the determination of M4 to M9, methanol was used as precipitant.

In the process of determining the concentration of berberine in rat plasma after intravenous administration, plasma samples collected from the first five time-points exceeded the highest concentration of berberine (100 ng/mL). Thus, dilution procedure was conducted. 10 μL of this plasma sample was diluted with 140 μL blank plasma and 50 μL of the diluted plasma sample was transferred to a new tube and treated followed the sample processing as mentioned above. This dilution procedure was validated with spiked samples at concentrations of 300, 750, and 1,350 ng/mL in quadruplicate. Additionally, the plasma concentration of M7 for several samples also exceeded the highest concentration. These samples were repeatedly analyzed using 25 μL of sample diluted with 25 μL blank plasma. This dilution procedure was also validated with spiked samples at concentrations of 500, 700, and 900 ng/mL in quadruplicate. The dilution factors for berberine and M7 were 15 and 2, respectively.

### Analysis of Berberine and Its Nine Metabolites in Rat Biological Samples

The LC-MS/MS system consisted of a Waters ACQUITY^TM^ I-Class series UPLC system and an AB SCIEX 5500 QTRAP triple quadrupole mass spectrometer operated in positive ESI mode. Multiple reaction monitoring (MRM) mode was applied for quantitation of berberine and its metabolites. The instrument parameters included an ion spray voltage of 5.5 kV, temperature of 500 °C, curtain gas of 30 psi and collision gas of 10 psi. Table 1 shows the MS parameters of the analytes and IS. Data acquisition and quantitation were conducted with Analyst version 1.6.2 (Applied Biosystems/MDS SCIEX).

**TABLE 1:** Mass scan method parameters of analytes and IS.

Analytes	Ion pairs (*m/z*)	DP (V)	CE (V)	EP (V)	CEP (V)
Berberine	336.2–292.2	30	40	10	20
M1	322.1–307.3	30	40	10	20
M2	324.1–280.2	30	40	10	20
M3	338.2–294.3	30	40	10	20
M4	514.1–338.3	70	36	10	9
M5	418.2–338.3	20	28	10	9
M6	498.2–322.3	12	36	10	9
M7	498.2–322.2	12	35	10	9
M8	404.2–324.1	20	30	10	9
M9	500.2–324.2	18	38	10	20
IS	356.2–192.1	30	40	10	20

CE, collision energy; CXP, collision cell exit potential; DP, declustering potential; EP, entrance potential.

Chromatographic separation was accomplished by using an ACQUITY UPLC BEH-C18 (1.7 μm, 2.1 × 50 mm) with an ACQUITY UPLC BEH-C18 guard cartridge (1.7 μm, 2.1 × 5 mm) at room temperature with a flow rate of 0.3 mL/min. The mobile phase was composed of 2 mM ammonium formate/formic acid (99.9:0.1, *v/v*, eluent A) and acetonitrile (eluent B) with gradient elution. Due to the fact that the polarity of phase II metabolites (M4–M9) was much greater than that of berberine and its phase I metabolites (M1–M3), two sets of mobile phase gradient elution programs were applied in this study. For the quantitative determination of berberine, M1, M2 and M3, gradient elution program one was used: 0–0.5 min, 28% B; 0.5–2.3 min, 28–62% B; 2.3–2.5 min, 62% B; 2.5–2.6 min, 62–95% B; 2.6–3.8 min, 95% B; 3.8–3.9 min, 95–28% B; 3.9–5.0 min, 28% B, and the switching valve directed the flow eluting between 0.5 and 3.0 min into the mass spectrometer and the remainder to a waste container. For the quantitative determination of M4 to M9, gradient elution program two was used: 0–4.5 min, 10–14% B; 4.5–5.5 min, 14–50% B; 5.5–6.0 min, 50–95% B; 6.0–7.0 min, 95% B; 7.0–7.1 min, 95–10% B; 7.1–8.0 min, 10% B, and the switching valve directed the flow eluting between 0.5 and 7.0 min into the mass spectrometer and the remainder to a waste container.

Method validation procedure was carried out according to the FDA guidance and selectivity, accuracy, precision, linearity, recovery, matrix effect and stability were assayed. Detailed information about the method validation could be found in the Supplementary Material.

### Pharmacokinetics and Excretion Study

Pharmacokinetic parameters, including area under plasma concentration-time cure (AUC), the peak plasma concentration (C_max_), time for peak plasma concentration (T_max_), elimination half-life (t_1/2_), apparent volume of distribution (Vz/F), apparent clearance (CLz/F) and mean residence time (MRT), were calculated based on a non-compartmental model with DAS 2.0 software (Shanghai, China) and compared using one-way analysis of variance. The AUC was calculated by the trapezoidal rule and C_max_ and T_max_ were obtained directly from the plasma profiles. The elimination rate constant (k_e_) was calculated from the slope of the terminal phase of the plasma concentration-time plot and t_1/2_ was calculated as 0.693/k_e_. Vz/F was calculated using the relationship, Vz/F = dose/ke·AUC_0–∞_. CLz/F was calculated from the relationship, CLz/F = dose/AUC_0–∞_. All pharmacokinetic and excretion parameters were expressed as mean ± SD. Statistical comparison was performed by one-way analysis of variance and a *p* value <0.05 or 0.01 was considered statistically significant.

## Results

### Method Validation

**FIGURE 2:**
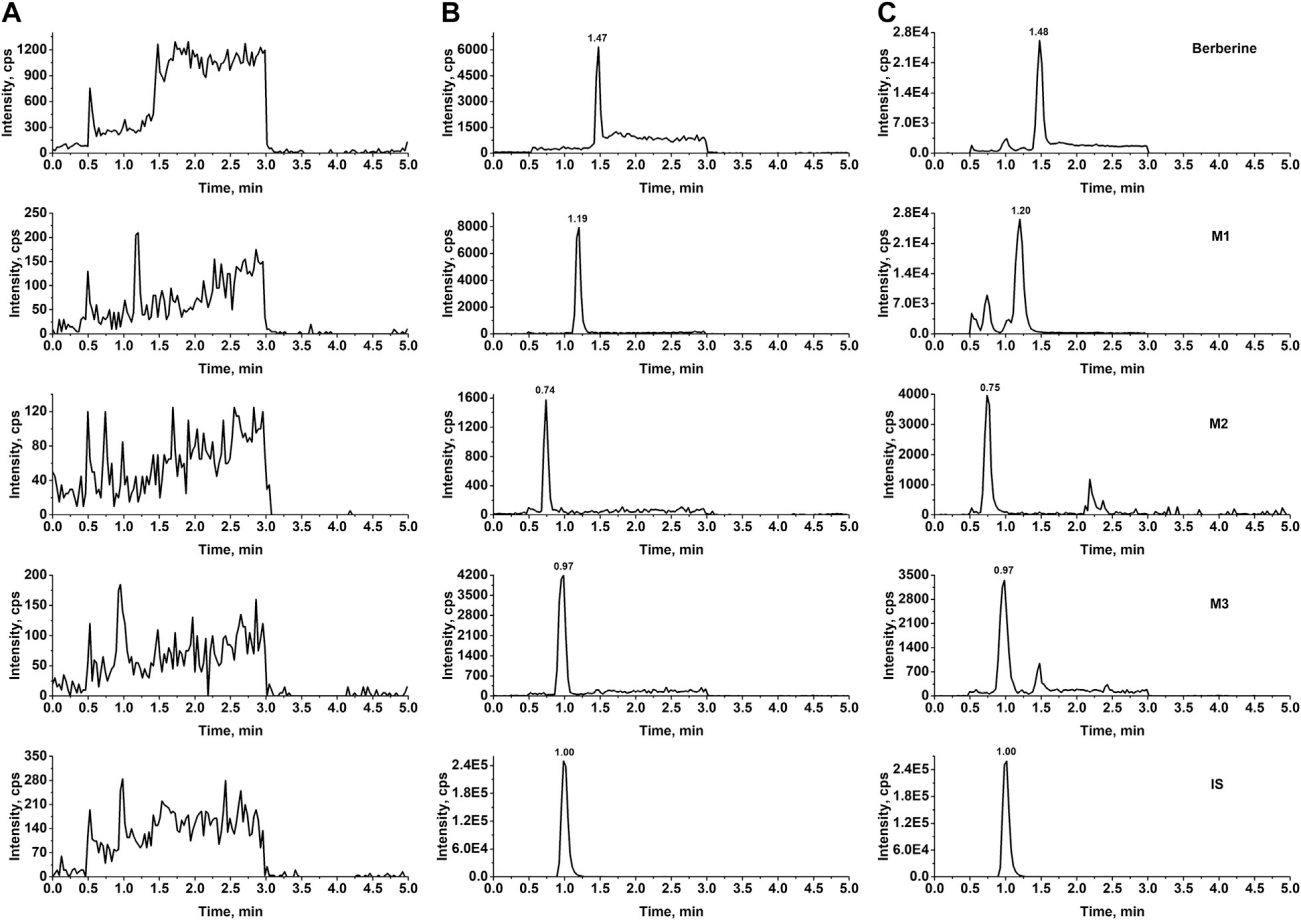
Typical MRM chromatograms of berberine, M1, M2, and M3 from rat plasma **(A)** Blank rat plasma **(B)** blank plasma samples spiked with standard substance (0.500 ng/mL) and IS **(C)** real plasma samples obtained from pharmacokinetics study.

**FIGURE 3:**
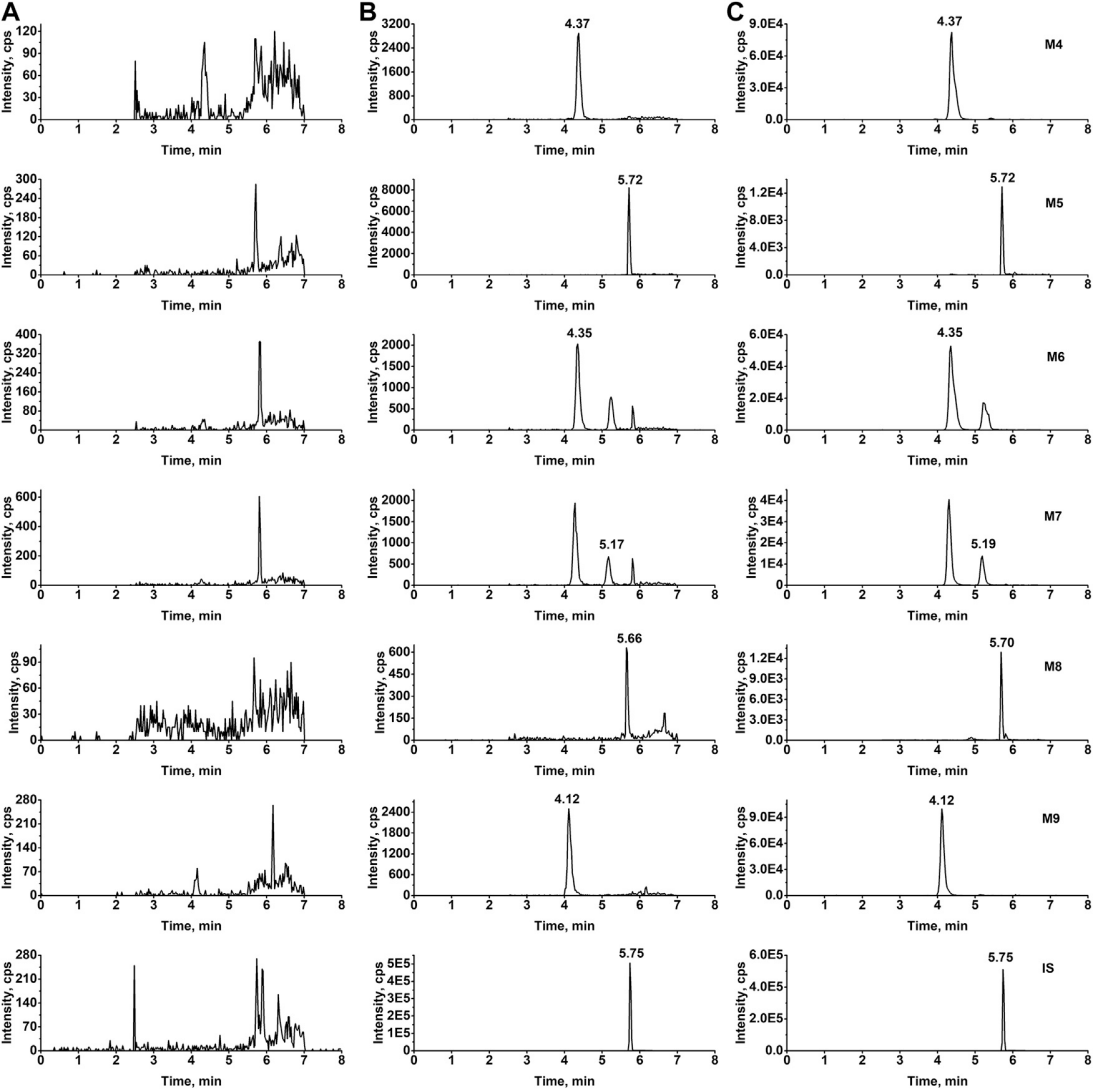
Typical MRM chromatograms of M4 to M9 from rat plasma **(A)** Blank rat plasma **(B)** blank plasma samples spiked with standard substance (1.00 ng/mL) and IS **(C)** real plasma samples obtained from pharmacokinetics study.

In our study, two sets of mobile phase gradient elution were applied. The first gradient elution was used for the determination of berberine, M1, M2, M3 and the total run time was 5.0 min. The retention times of berberine, M1, M2, M3 and IS were 1.5, 1.2, 0.75, 0.97, and 1.01 min, respectively. For the determination of M4 to M9, the second gradient elution was applied and the total run time was 8.0 min. The retention times of M4 to M9, and IS were 4.4, 5.7, 4.3, 5.2, 5.7, 4.2, and 5.8 min, respectively. Figures 2, 3 show representative chromatograms of a bank plasma, a blank plasma spiked with berberine and its nine metabolites, and a real plasma sample from pharmacokinetics study. No endogenous interfering peaks could be found at retention times of analytes and IS. Calibration curves all showed good linearity over concentration range of 0.500–100 ng/mL (berberine and M1 to M3) and 1.00–500 ng/mL (M4 to M9), and all correlation coefficients (*r*) were greater than 0.9981 in all analytes. The lower limit of quantifications (LLOQs) of berberine and M1 to M3 were 0.500 ng/mL and the LLOQs of M4 to M9 were 1.00 ng/mL with both precision and accuracy were less than 20.0%. The relative standard deviation (RSD) values for intra-and inter-day analysis were less than 9.4% and the relative error (RE) values were in the range of −11.4 to 13.8%. The matrix effects of all analytes were within 92.5–109% and the extraction recovery ranged from 69.8 to 94.7%. The stability of berberine and its nine metabolites was evaluated by analyzing quality control (QC) samples at low and high concentration levels. The RSD values ranged from 0.4 to 8.5% and the RE values ranged from −13.3 to 13.5%, which indicated that all samples were stable for 4 h at room temperature, after three freeze-thaw cycles, at −80 °C for 21 days, and post-preparative QC samples stored at 10 °C for 12 h were also stable. Validation of dilution procedure was conducted with spiked samples in quadruplicates. The dilution procedure showed excellent accuracy (RE values ranged from −3.6 to 7.0%) and precise (RSD < 9.4%). Detailed results of method validation were showed in Supplementary Tables S1–S4.

### Pharmacokinetics Study

**FIGURE 4:**
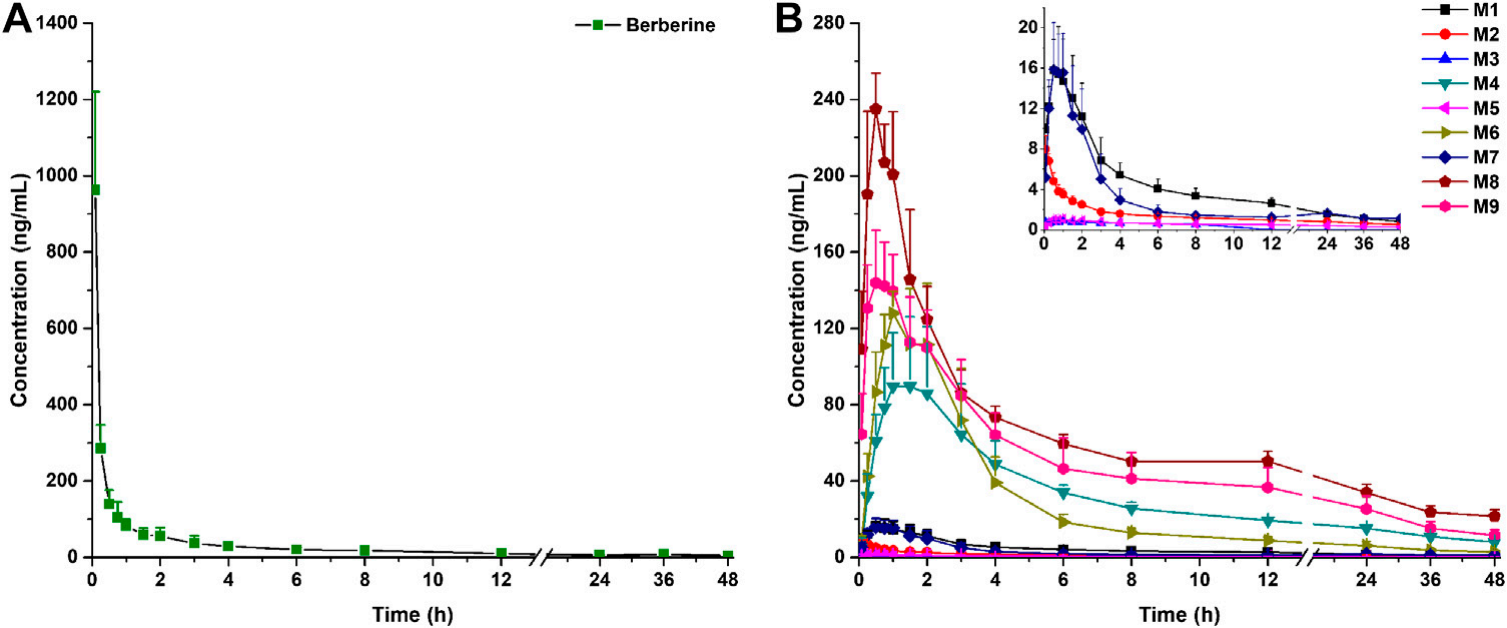
Mean plasma concentration-time curves of berberine **(A)** and its nine metabolites **(B)** in rats following intravenous administration of 4.0 mg/kg berberine (Mean ± SD, *n* = 6). The inserted scale-adjusted figure in **(B)** showed the mean plasma concentration-time curves of M1, M2, M3, M5, and M7.

**FIGURE 5:**
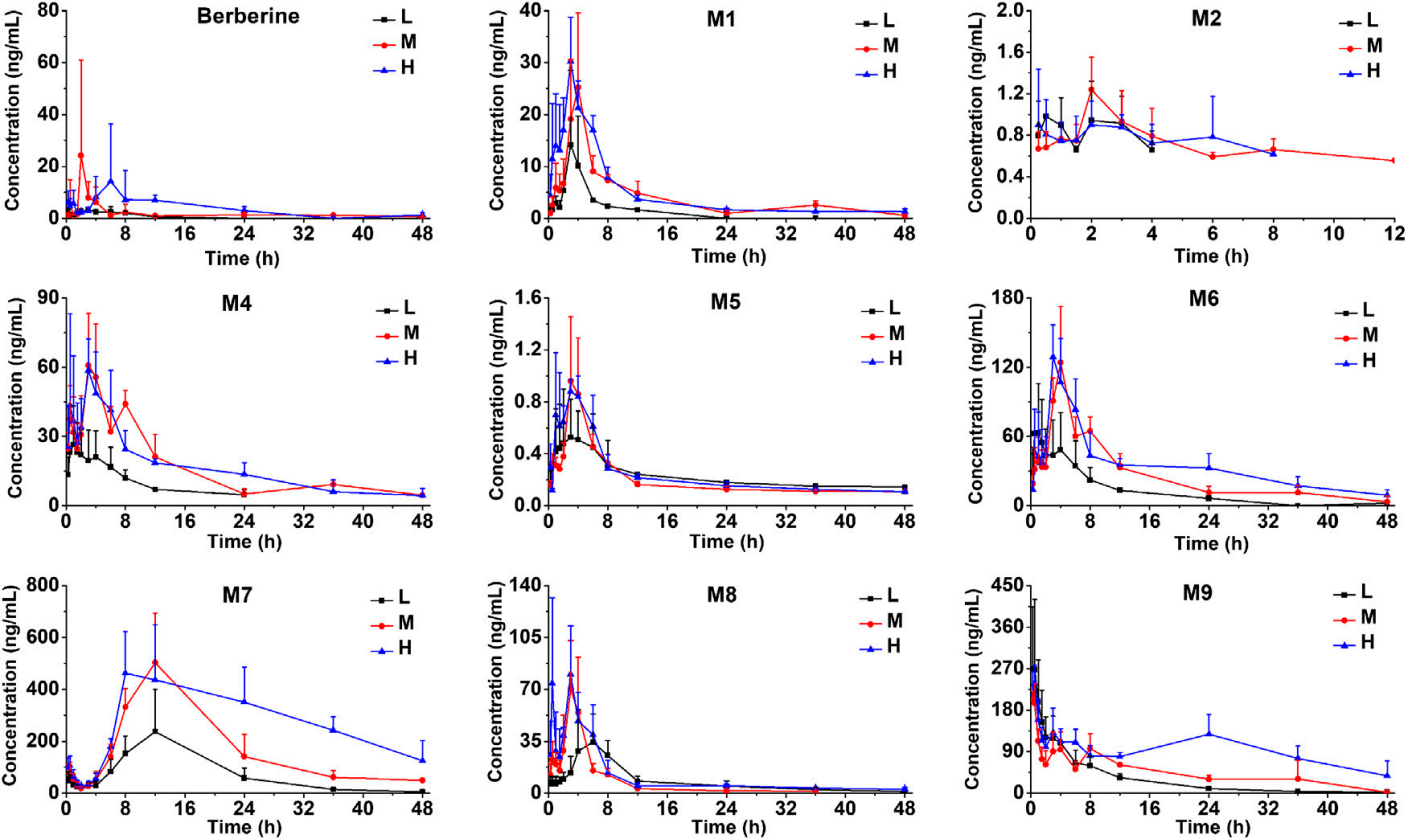
Mean plasma concentration-time curves of berberine and its nine metabolites in rats following oral administration of berberine at a single dose of 48.2 mg/kg (low, L), 120 mg/kg (medium, M) or 240 mg/kg (high, H) (Mean ± SD, *n* = 6).

The LC-MS/MS method was successfully applied to a pharmacokinetics study of berberine and its nine metabolites following a single oral administration of either 48.2, 120, or 240 mg/kg berberine and a single intravenous administration of berberine at the dose of 4.0 mg/kg. Pharmacokinetic parameters are listed in Table 2 and the mean plasma concentration-time curves are presented in Figures 4, 5.

**TABLE 2:** Pharmacokinetic parameters of berberine and its nine metabolites in rats after intravenous or oral administration of berberine.

Analytes	Dose (mg/kg)	The route of administration	AUC_0–48 h_ (mg/L * h)	AUC_0–∞_ (mg/L * h)	C_max_ (ng/mL)	T_max_ (h)	t_1/2z_ (h)	Vz/F (L/kg)	CLz/F (L/h/kg)	MRT_0–48 h_ (h)	MRT_0–∞_ (h)
Berberine	4.0	i.v	839.00 ± 102.00	963.00 ± 134.00	963.00 ± 258.00	—	23.60 ± 6.53	0.71 ± 0.18	0.021 ± 0.003	9.95 ± 2.70	20.3 ± 4.03
	48.2	p.o	22.50 ± 6.88	31.40 ± 3.01	4.11 ± 0.53	2.75 ± 2.95	5.74 ± 4.60	12.20 ± 8.71	1.54 ± 0.14	4.76 ± 1.02	9.51 ± 4.97
	120	p.o	81.30 ± 16.30	99.00 ± 12.10	32.60 ± 30.50	1.88 ± 1.03	21.20 ± 9.85	38.70 ± 21.60	1.23 ± 0.15	12.30 ± 5.98	24.20 ± 9.79
	240	p.o	166.00 ± 111.00	282.00 ± 122.00	18.00 ± 14.80	2.62 ± 2.86	17.10 ± 14.10	27.20 ± 29.20	0.97 ± 0.39	16.30 ± 6.63	22.40 ± 12.10
M1	4.0	i.v	123.00 ± 21.20	144.00 ± 18.70	16.60 ± 4.11	0.67 ± 0.20	20.00 ± 4.73	4.11 ± 1.21	0.14 ± 0.017	13.40 ± 1.06	24.30 ± 4.21
	48.2	p.o	82.90 ± 46.10	97.40 ± 44.20	16.60 ± 11.40	2.60 ± 0.89	7.32 ± 3.13	6.14 ± 3.87	0.58 ± 0.18	6.62 ± 1.75	9.88 ± 3.28
	120	p.o	167.00 ± 61.60*	177.00 ± 73.10	29.10 ± 13.70	3.50 ± 0.55	6.18 ± 2.95	6.30 ± 2.21	0.76 ± 0.26	9.08 ± 1.14	12.20 ± 2.94
	240	p.o	226.00 ± 23.20*	232.00 ± 28.60	31.30 ± 6.58	2.50 ± 1.00	8.99 ± 5.49	13.20 ± 6.69	1.05 ± 0.14	11.00 ± 1.78	14.80 ± 2.83
M2	4.0	i.v	41.70 ± 8.36	68.20 ± 17.20	7.96 ± 1.36	0.083 ± 0.00	30.90 ± 11.80	12.80 ± 1.90	0.31 ± 0.078	13.60 ± 3.05	40.30 ± 15.00
	48.2	p.o	3.24 ± 1.98	4.87 ± 3.04	1.18 ± 0.21	1.17 ± 0.76	2.16 ± 1.35	32.30 ± 2.62	13.50 ± 8.32	1.91 ± 0.84	3.67 ± 1.87
	120	p.o	5.15 ± 2.35	12.00 ± 7.44	1.26 ± 0.27	2.20 ± 0.45	6.18 ± 6.35	75.90 ± 40.00	13.60 ± 7.40	3.25 ± 1.62	8.69 ± 8.66
	240	p.o	4.81 ± 2.43	8.39 ± 4.93	1.17 ± 0.31	1.08 ± 0.88	4.34 ± 2.63	176.00 ± 19.10	35.90 ± 19.60	2.96 ± 0.86	6.91 ± 3.38
M3	4.0	i.v	4.98 ± 2.02	14.10 ± 4.55	0.94 ± 0.21	1.06 ± 0.65	11.50 ± 4.23	23.40 ± 3.36	1.53 ± 0.41	3.39 ± 1.20	16.80 ± 5.89
M4	4.0	i.v	983.00 ± 136.00	1,275.00 ± 111.00	92.80 ± 35.00	1.10 ± 0.22	25.20 ± 4.05	0.12 ± 0.021	0.003 ± 0.00	15.40 ± 1.18	31.50 ± 6.06
	48.2	p.o	246.00 ± 95.20	269.00 ± 99.80	31.70 ± 14.00	2.83 ± 1.17	5.72 ± 2.40	1.70 ± 0.85	0.21 ± 0.082	6.69 ± 2.11	9.84 ± 4.31
	120	p.o	681.00 ± 264.00**	721.00 ± 281.00	62.60 ± 20.90	4.75 ± 2.22	7.42 ± 3.03	1.83 ± 0.49	0.18 ± 0.06	9.74 ± 2.63	12.40 ± 3.36
	240	p.o	764.00 ± 128.00**	833.00 ± 177.00	76.40 ± 9.68	2.80 ± 2.36	11.70 ± 3.78	4.81 ± 0.89	0.30 ± 0.073	13.50 ± 3.86	17.20 ± 7.09
M5	4.0	i.v	21.80 ± 2.99	40.80 ± 8.24	1.10 ± 0.27	1.00 ± 0.55	45.00 ± 14.20	6.31 ± 1.09	0.10 ± 0.024	20.00 ± 0.74	66.80 ± 17.40
	48.2	p.o	5.22 ± 4.65	6.28 ± 5.47	0.58 ± 0.20	3.00 ± 0.71	7.89 ± 6.26	94.70 ± 34.40	11.10 ± 4.53	8.09 ± 5.00	14.60 ± 10.70
	120	p.o	9.70 ± 1.08	13.30 ± 3.02	0.99 ± 0.46	3.25 ± 0.50	28.40 ± 14.30	350.00 ± 134.00	9.45 ± 2.43	15.90 ± 1.47	42.40 ± 13.90
	240	p.o	10.60 ± 2.12	14.20 ± 4.80	0.94 ± 0.14	3.33 ± 2.52	23.40 ± 13.10	536.00 ± 172.00	18.70 ± 7.59	14.30 ± 2.56	32.30 ± 6.94
M6	4.0	i.v	659.00 ± 108.00	739.00 ± 129.00	134.00 ± 18.20	1.17 ± 0.41	20.00 ± 4.19	0.16 ± 0.038	0.006 ± 0.001	9.69 ± 1.15	17.60 ± 4.29
	48.2	p.o	535.00 ± 148.00	572.00 ± 146.00	85.10 ± 36.50	2.33 ± 1.47	6.53 ± 4.60	0.86 ± 0.67	0.092 ± 0.021	7.30 ± 3.44	9.93 ± 5.31
	120	p.o	1,159.00 ± 264.00**	1,197.00 ± 278.00	130.00 ± 42.30	3.83 ± 0.41	7.40 ± 2.37	1.07 ± 0.28	0.10 ± 0.022	10.90 ± 2.32	13.10 ± 4.09
	240	p.o	1,621.00 ± 179.00**^#^	1889.00 ± 196.00	138.00 ± 28.30	3.25 ± 0.50	18.00 ± 4.51	3.24 ± 1.04	0.13 ± 0.01	15.90 ± 3.06	22.20 ± 4.19
M7	4.0	i.v	90.10 ± 18.80	277.00 ± 118.00	17.40 ± 3.91	0.70 ± 0.27	102.00 ± 77.20	1.96 ± 0.78	0.016 ± 0.005	13.40 ± 2.11	129.00 ± 112.00
	48.2	p.o	3,715.00 ± 906.00	3,759.00 ± 885.00	238.00 ± 161.00	11.00 ± 2.00	7.03 ± 1.87	0.14 ± 0.054	0.014 ± 0.0030	15.20 ± 3.05	16.00 ± 3.53
	120	p.o	7,791.00 ± 1,478.00**	8,902.00 ± 927.00	503.00 ± 190.00	12.00 ± 0.00	13.50 ± 3.47	0.27 ± 0.082	0.013 ± 0.0020	15.60 ± 2.61	21.80 ± 5.66
	240	p.o	13,362.00 ± 1,062.00**^#^	17,954.00 ± 3,789.00	591.00 ± 102.00	13.00 ± 7.57	21.00 ± 6.58	0.40 ± 0.006	0.014 ± 0.0030	22.20 ± 3.05	32.90 ± 9.43
M8	4.0	i.v	2095.00 ± 108.00	2,816.00 ± 417.00	238.00 ± 18.00	0.54 ± 0.25	26.60 ± 7.40	0.054 ± 0.009	0.001 ± 0.001	16.20 ± 1.00	38.20 ± 8.99
	48.2	p.o	149.00 ± 52.90	155.00 ± 50.60	40.30 ± 23.50	2.80 ± 0.84	2.50 ± 0.86	1.30 ± 0.64	0.36 ± 0.14	4.45 ± 1.13	5.10 ± 1.60
	120	p.o	311.00 ± 121.00	320.00 ± 127.00	74.10 ± 36.90	3.40 ± 0.55	5.48 ± 2.69	3.08 ± 1.61	0.42 ± 0.13	5.97 ± 1.37	7.19 ± 2.17
	240	p.o	601.00 ± 186.00**^#^	671.00 ± 151.00	98.20 ± 19.50*	1.06 ± 1.29	20.50 ± 5.82	11.40 ± 5.28	0.37 ± 0.077	10.70 ± 2.71	20.30 ± 11.90
M9	4.0	i.v	1,547.00 ± 313.00	1976.00 ± 353.00	154.00 ± 19.90	0.62 ± 0.21	24.00 ± 7.27	0.071 ± 0.023	0.002 ± 0.00	15.10 ± 1.19	28.80 ± 7.54
	48.2	p.o	1,476.00 ± 327.00	1,513.00 ± 335.00	282.00 ± 145.00	0.50 ± 0.35	7.97 ± 2.47	0.41 ± 0.19	0.034 ± 0.0070	8.40 ± 2.66	9.34 ± 3.05
	120	p.o	2,129.00 ± 749.00	2,154.00 ± 754.00	213.00 ± 33.10	0.30 ± 0.11	7.52 ± 1.27	0.66 ± 0.23	0.061 ± 0.019	14.30 ± 4.12	14.80 ± 4.08
	240	p.o	4,417.00 ± 966.00**^#^	5,956.00 ± 2,168.00	252.00 ± 25.70	0.40 ± 0.14	25.30 ± 16.80	1.46 ± 0.62	0.045 ± 0.015	20.60 ± 2.31	37.80 ± 22.10

Compared to 48.2 mg group, *p < 0.05, **p < 0.01; compared to 120 mg group, ^#^p < 0.01.

After intravenous administration, the plasma concentration-time curve of berberine showed a rapid decline from 963 ± 258 to 9.82 ± 2.79 ng/mL after 12 h post-dose. Within 1.5 h after intravenous administration, all the nine metabolites were formed and reached their peak plasma concentrations. After oral administration, plasma concentrations for M3 at different time points were all below the LLOQ (0.500 ng/mL), thus, no plasma concentration-time curve and pharmacokinetic parameters for M3 were obtained in this study. Plasma concentrations for M2 after 12 h were all below the LLOQ (0.500 ng/mL), thus, the plasma concentration-time curve for M2 was displayed from 0 to 12 h. Following oral administration of berberine at the dose of 48.2, 120, and 240 mg/kg, the AUC_0–48 h_ for berberine, M1, M6, M7, M8, and M9 increased with the dose. As shown in Supplementary Figure S1, dose proportionality was observed for berberine, M1, M6, M7, M8, and M9 in terms of dose and AUCs (*r* ^2^ ranged from 0.8823 to 0.9994). As for M2, M4, and M5, no significant dose proportionality was observed. Additionally, C_max_ values of the berberine and its metabolites was not dose-dependent. This nonlinear pharmacokinetic behavior might be caused by the saturation of metabolism enzymes or carrier-mediated systems involved in the processes of drug absorption, distribution, biotransformation, and excretion. A comparison of the AUC_0–∞_ values of berberine given either orally or intravenously revealed that the absolute bioavailability of berberine was only 0.37 ± 0.11%. After oral administration, AUC_0–∞_ values of phase II metabolites (except for M5) were higher than that of phase I metabolites, indicating that phase II metabolites were the major exposure forms of berberine *in vivo*. The Vz/F of berberine was found to be 12–38 L/kg, which was exceeded the total body water in rats (0.15 L/kg for a body weight of 0.25 kg), suggesting that berberine may be well distributed in the extravascular fluids. After oral administration, the T_max_ values for M7 were 11–13 h, indicating that the biotransformation of berberine into M7 was processed gradually. Conversely, T_max_ values for other metabolites were all less than 4.75 h and the short T_max_ indicated that berberine could be rapidly metabolized into various metabolites.

### Excretion Study

The LC-MS/MS method was also applied for the excretion study of berberine and its nine metabolites following a single oral administration of berberine (48.2 mg/kg) to rats. Detailed excretion data of berberine and its nine metabolites was showed in Supplementary Tables S5–S7. The excretion amount, excretion rate and accumulative excretion were calculated according to the equations.

$$\begin{array}{l} \text{Excretion}\;\text{amount}\left(\text{ng}\right)\;\text{at}\;\text{time}\;t\left(\Delta U\right)=\left[\text{Concentration}\;\text{of}\;\text{analyte}\;\left(\text{ng}/\text{ml}\right)\;\text{at}\;t\right]\times \text{Total}\;\text{biosample}\;\text{volume}\;\left(\text{ml}\right),\\ \text{Excretion}\;\text{rate}\left(\text{ng}/\text{h}\right)=\text{Excretion}\;\text{amount}\left(\Delta U\right)/\text{Time}\;\text{interval}\left(\Delta t\right),\\ \text{Accumulative}\;\text{excretion}\;\text{up}\;\text{to}\;\text{time}\;t\left(\%\right)=100\times \left[\sum t\;\text{excretion}\;\text{amount}\left(\Delta U\right)t\right]/\text{Total}\;\text{ingested}. \end{array}$$

**FIGURE 6:**
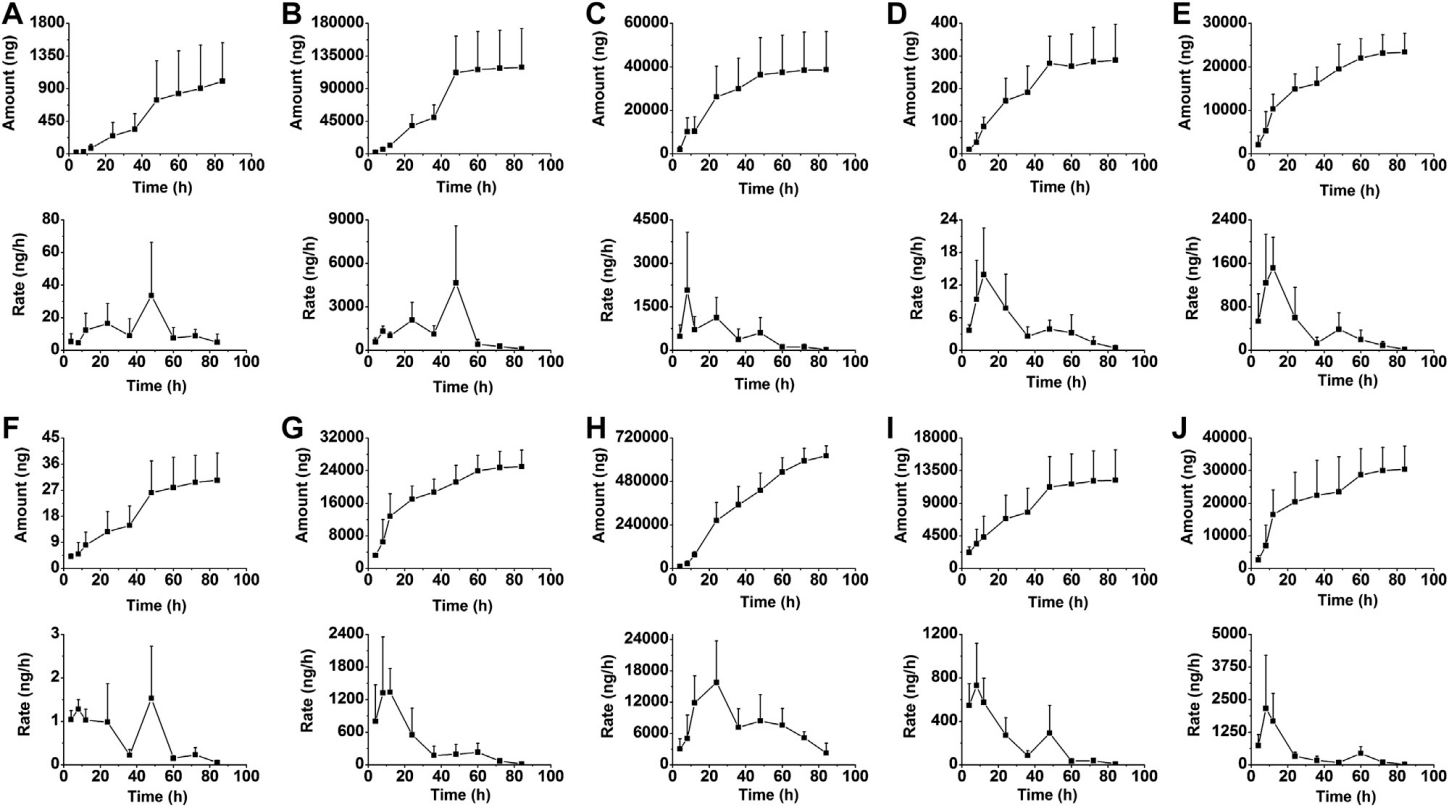
The mean urinary excretion accumulative amount-time profiles and excretion rate-time profiles of berberine **(A)**, and M1 **(B)** to M9 **(J)** in rats following oral administration of berberine at a single dose of 48.2 mg/kg (Mean ± SD, *n* = 6).

**FIGURE 7:**
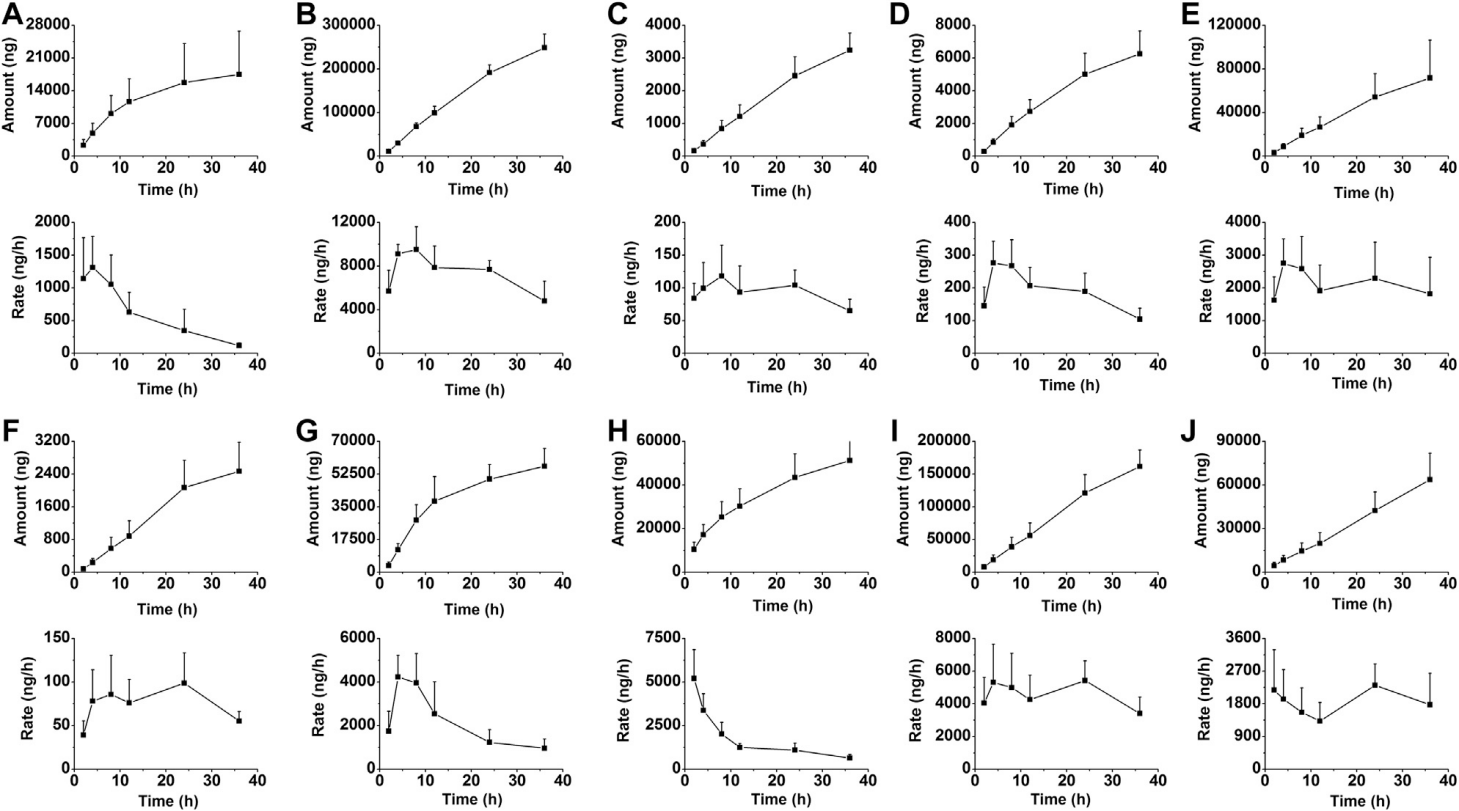
The mean biliary excretion accumulative amount-time profiles and excretion rate-time profiles of berberine **(A)**, and M1 **(B)** to M9 **(J)** in rats following oral administration of berberine at a single dose of 48.2 mg/kg (Mean ± SD, *n* = 6).

**FIGURE 8:**
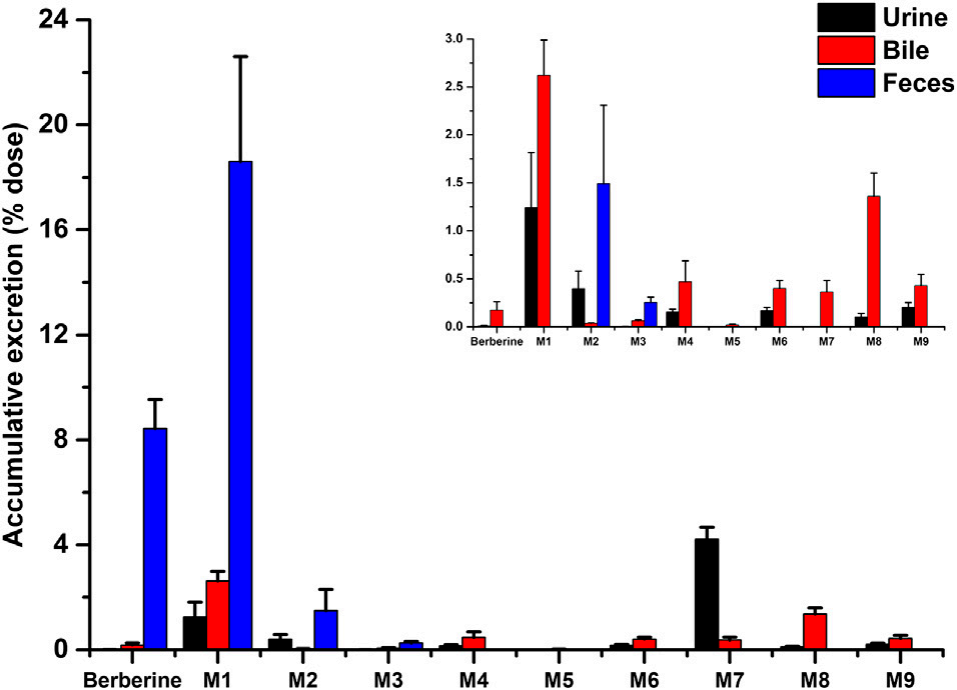
Accumulative excretion of berberine and its nine metabolites in urine and bile after a single oral administration of 48.2 mg/kg berberine to rats.

The mean urinary and biliary excretion accumulative amount-time profiles and excretion rate-time profiles of berberine and its nine metabolites are showed in Figures 6, 7. As shown in Figure 8, after oral administration, the cumulative urinary excretion of berberine and its nine metabolites (M1 to M9) for 84 h were 0.0101, 1.24, 0.399, 0.00284, 0.153, 0.000245, 0.169, 4.21, 0.102, and 0.204%, respectively. The cumulative biliary excretion of berberine and its nine metabolites (M1 to M9) for 36 h were 0.174, 2.62, 0.0339, 0.0623, 0.470, 0.0200, 0.401, 0.365, 1.36, and 0.430%, respectively. In the meanwhile, berberine and its three phase I metabolites (M1 to M3) were also detected in rat feces and the recoveries of them were 8.43, 18.6, 1.49, and 0.254% for 84 h. The total excretion recovery of berberine and its nine metabolites from urine, bile and feces were 8.61, 22.5, 1.92, 0.319, 0.623, 0.0202, 0.570, 4.58, 1.46, and 0.634%, respectively and the total excretion recovery was 41.2%.

## Discussion

The metabolism of berberine in rats had been widely reported. In our previous study conducted by UPLC-Q-TOF-MS, 45 metabolites were detected in rat plasma (Wang et al., 2017a). Among all the 45 metabolites, berberrubine, demethyleneberberine and jatrorrhizine were the most abundant phase I metabolites and M4 to M9 were the phase II metabolites with high contents. Thus, they were selected and studied for pharmacokinetic and excretion studies after administration of berberine. Thalifendine was also detected as a main metabolite of berberine, however, the plasma concentration of it was very low in our preliminary study. Meanwhile, dihydroberberine was not detected in our previous study, even though it had been proved to be a main important metabolite of berberine in gut microbiota. This might be caused by the fact that GC-MS could be more suitable for the detection of gut microbiota-metabolites, while only LC-MS method was used in our studies. Taken together, M1 to M9 were determined in this study. In order to provide an in-depth investigation of the pharmacokinetics and excretion of berberine, an accurate and reliable LC-MS/MS method was developed and validated for the determination of berberine and its metabolites in rat biosamples in this study. Due to the fact that the polarity of phase I and phase II metabolites varies a lot, two sets of mobile phase gradient elution programs were used in our LC-MS/MS method. Berberine and three phase I metabolites were analyzed with gradient elution program 1, and six phase II metabolites were analyzed with gradient elution program 2. Additionally, our preliminary study found that the plasma concentrations of M5 were all lower than 2.00 ng/mL after oral administration of berberine, thus, the linear range of M5 was adjusted to 0.100–50.0 ng/mL, and partial validation was performed and detailed results were showed in Supplementary Material. Our previous metabolism study of berberine showed that some metabolites were isomers, such as thalifendine (*m/z* 322.1) vs berberrubine (M1, *m/z* 322.1), thalifendine-10-*O-β*-D-glucuronide (M6, *m/z* 498.2) vs berberrubine-9-*O-β*-D-glucuronide (M7, *m/z* 498.2), jatrorrhizine (M3, *m/z* 338.2) vs columbamine (*m/z* 338.2) et al. Under our chromatographic condition, thalifendine and berberrubine (M1) could be separated (Supplementary Figure S2), and M6 and M7 were separated well; however, jatrorrhizine (M4) and columbamine could not be separated. Thus, the plasma concentrations of jatrorrhizine were the sum of jatrorrhizine and columbamine. Similar situations occurred with the phase II conjugations of jatrorrhizine.

In the pharmacokinetic study, berberine chloride was used for the oral administration. Due to the fact that the solubility of berberine chloride was extreme low that berberine sulfate (a widely used veterinary drug for injection) was used for intravenous administration. The absolute bioavailability of berberine was found to be 0.37 ± 0.11%, which was consistent with the reported results (Liu Y. T. et al., 2010; Chen et al., 2011). There were three reasons may account for the extremely low bioavailability of berberine. Firstly, berberine (pKa = ∼15) (Spinozzi et al., 2014) was mostly in the ionized form at physiological condition, thus, only a few could be absorbed after oral administration. Secondly, it had been reported that berberine was a substrate of P-gp and the bioavailability of berberine was greatly limited by this ATP-dependent multidrug transporter (Chen et al., 2011). Thirdly, berberine could undergo extensive first-pass metabolism in both intestine and liver. Even though previous studies demonstrated that intestinal flora could not significantly metabolize berberine, recent studies revealed that dihydroberberine and butyrate were metabolites of berberine generated by gut microbiota in rats (Feng et al., 2018).

After oral administration, the AUC_0–48 h_ value of berberrubine (M1) was much higher than those of demethyleneberberine (M2) and jatrorrhizine (M3), indicating that berberrubine was the main phase I metabolite. Additionally, the glucuronide and sulfate conjugations of M2 and M3, could be detected with T_max_ less than 4.75 h, indicating that M2 and M3 could be metabolized into phase II conjugations rapidly. However, T_max_ for M7 was found to be 11.00–13.00 h, suggesting that the glucuronidation of berberrubine (M1) was processed gradually. This might be another reason that berberrubine was detected with higher AUC_0–48 h_. According to the AUC_0–48 h_ values of both phase I and phase II metabolites, the metabolic levels of phase II metabolites were much higher than those of phase I metabolites, indicating that phase II metabolic pathways were the major metabolic routes. Compared with sulfation metabolites (M5 and M8), glucuronidation metabolites (M4 and M9) showed much higher values of AUC_0–48 h_ and C_max_, suggesting that glucuronidation was the predominant metabolic pathway for berberine following oral administration.

The total exposure amount (AUC_0–48 h_) with the order from large to small was M8 > M9 > M6 > M4 > M7 > M1 > M2 > M5 > M3 when berberine was given *i.v*. When berberine was given *i.g.*, the total exposure amount (AUC_0–48 h_) with the order from large to small was M7 > M9 > M6 > M4 > M8 > M1 > M2 > M5. These results indicated that the metabolic extent of berberine transformed to metabolites, especially glucuronidation metabolites (M7, M9, M6, and M4) after oral dose was more than that after intravenous dose. Thus, in addition to metabolism in liver, the intestinal flora and microsomes also play vital roles in the metabolism of berberine. As for M7, two maximum concentrations were observed at 0.25 and 12 h, respectively. The appearance of a secondary plasma peak may be due to an enterohepatic circulation, namely, M7, which was produced in liver *via* the glucuronidation of berberrubine was secreted into bile and subsequently released into the small intestine, where M7 was further hydrolyzed to berberrubine by enterobacteria and reabsorbed back into circulation and subsequently glucuronidated to produce M7 in the liver. Even though the double-absorption peak phenomenon for other phase II metabolites was not so significant, it was still observed, especially when berberine was orally administrated at the dose of 120 mg/kg. The enterohepatic circulation of berberine metabolites was also reported by Zou et al. (Zuo et al., 2006). Their study revealed that intestinal flora played a significant role in the enterohepatic circulation of berberine metabolites and the pharmacokinetics of berberine and its metabolites significantly differed between conventional and pseudo germ-free rats. This result was also supported by the data of Alolga et al. (Alolga et al., 2016) who reported that the pharmacokinetic profile differences of berberine observed between Africans and Chinese was partly attributable to the variations in intestinal flora and its corresponding metabolic capacity.

As we can see from the excretion results, berberine was mainly excreted in feces. At physiological condition, berberine was mostly in the ionized form and only a few could be absorbed from intestine. Thus, berberine detected in feces might be from the original unabsorbed drugs after oral administration. Meanwhile, 18.6% of the berberine was excreted in feces as berberrubine (M1). The accumulative biliary excretions of berberrubine (M1) and berberrubine-9-*O-β*-D-glucuronide (M7) were 2.62 and 0.356%, respectively, and the sum of these two accumulative excretions (2.976%) was much lower than the fecal excretion of berberrubine (18.6%). These results indicated that most of the berberrubine excreted in feces may be derived from the unabsorbed berberine via demethylation metabolism. Until now, whether gastrointestinal tract could participate in the metabolism of berberine is still controversial. Some results suggested that berberine was stable in gastrointestinal tract (Zuo et al., 2006; Qiu et al., 2008), however, some investigators reported that metabolites such as berberrubine, demethyleneberberine, jatrorrhizine, dihydroberberine and butyrate were generated in the intestinal ecosystem (Li et al., 2014; Feng et al., 2018). The excretion results obtained in our study suggested that berberine could be metabolized into berberrubine in gastrointestinal tract either *via* the metabolism enzymes exist in gut wall or *via* gut microbiota.

## Conclusion

In summary, we present a comprehensive pharmacokinetics and excretion study of berberine and its nine metabolites in rats. To our knowledge, the pharmacokinetics and excretion results of berberine’s six phase II metabolites in rats after *i.g.* and *i.v.* administration were reported for the first time. The absolute bioavailability of berberine was very low due to insufficient absorption and extensive metabolism. Berberine, M1, M6, M7, M8, and M9 showed linear dynamics in dose range of 48.2–240 mg/kg. Phase II metabolites were the major circulating components in plasma. The total excretion of berberine and its metabolites in urine, bile and feces was 41.2% within 84 h. Berberine was mainly excreted in the form of berberrubine and berberrubine-9-*O-β*-D-glucuronide *via* fecal and renal excretion route, respectively. The present disposition studies of berberine in rats will be beneficial for both better understanding the clinical effects and further development of berberine. Meanwhile, due to the inter-species differences, further studies are still needed to extrapolate the pharmacokinetic findings to human.

## Data Availability Statement

The original contributions presented in the study are included in the article/Supplementary Material, further inquiries can be directed to the corresponding author.

## Ethics Statement

The animal study was reviewed and approved by Animal Ethics Committee of Tianjin University of Traditional Chinese Medicine.

## Author Contributions

XF and KW conducted the experiments, preformed the data analysis and wrote the manuscript. SC and LD preformed the data analysis. FQ designed and supervised this research.

## Funding

The project was supported by the Technology Major Project of China “Key New Drug Creation and Manufacturing Program” (2017ZX09301012-001 and 2017ZX09301005), Major State Basic Research Development Program of China (2014CB560706), and National Natural Science Foundation of China (82030116, 81430095 and 81703776).

## Conflict of Interest

The authors declare that the research was conducted in the absence of any commercial or financial relationships that could be construed as a potential conflict of interest.
