# Supplementary material for: Pharmacokinetics and Excretion of Berberine and Its Nine Metabolites in Rats
Source: Front Pharmacol. 2021 Jan 15;11:594852. doi: 10.3389/fphar.2020.594852 (PMC7874128; doi:10.3389/fphar.2020.594852)
Supplement: Supplementary file 1 [file datasheet1.pdf]

Supplementary information for

Pharmacokinetics and excretion of berberine and its nine metabolites in rats

1 Preparation of calibration standard samples and quality control samples

Standard stock solutions of berberine and its nine metabolites, and IS were separately prepared in dimethylsulfoxide (DMSO) at concentrations of 1.00 mg/mL. For berberine, M1, M2 and M3, appropriate dilutions of the stock solutions were made with acetonitrile-water (*v/v* = 1:1) to obtain a series of analytes working solutions (2.50, 5.00, 10.0, 25.0, 50.0, 100, 250 and 500 ng/mL). Quality control (QC) working solutions at concentrations of 7.50, 40.0 and 400 ng/mL were made from 1.00 mg/mL stock solution of analytes, respectively. For M4 to M9, stock solutions were diluted with methanol-water (*v/v* = 1:1) to obtain a series of analytes working solutions (5.00, 10.0, 25.0, 100, 250, 500, 1000 and 2500 ng/mL) and QC working solutions were at concentrations of 15.0, 200 and 2000 ng/mL. The concentration of IS was 5.00 ng/mL and for the determination of berberine, M1, M2 and M3, IS was prepared in acetonitrile, while for the determination of M4 to M9, IS was prepared in methanol. All solutions were kept at 4°C and brought to room temperature before uses in the preparation of calibration standard and QC samples.

Calibration standard samples were prepared by adding 50 μL of the blank plasma to 10 μL of the analytes working solutions contained in Eppendorf tubes, respectively. QC samples were prepared by the same procedure as above.

2 Method validation

The LC–MS/MS method was validated according to the FDA guidance. The selectivity of the method was evaluated by analyzing blank samples of rat plasma from six rats to check for interfering peaks of analytes and IS, respectively. Matrix effect was investigated using the post-extraction spiked samples at three QC levels prepared from the plasma of six different rats. The matrix factor in each matrix was calculated by comparing the peak areas of analytes and IS obtained from the post-extraction spiked samples to those from the QC working solutions at respective QC level. The accuracy and precision of the method were assessed by analyzing QC samples in six replicates at three QC levels through three validation batches. Accuracy was calculated as percentage deviation of the measured concentration from the nominal concentrationand expressed as relative error (RE). Intra- and inter-batch precisions were indicated by relative standard derivation (RSD). The extraction recoveries of the analytes and IS were determined at three QC levels by comparing the peak areas of the analytes and IS obtained from plasma samples with analytes spiked before extraction to that spiked after extraction.

The linearity of the method was assessed by analyzing calibration standard samples in duplicate at each concentration level over three consecutive batches. For berberine, M1, M2 and M3, the calibration standard samples concentrations were 0.50, 1.00, 2.00, 5.00, 10.0, 20.0, 50.0 and 100 ng/mL. For M4 to M9, the calibration standard samples concentrations were 1.00, 2.00, 5.00, 20.0, 50.0, 100, 200 and 500 ng/mL. Calibration curves (peak area ratio of the analytes to IS versus the

nominal concentration) were fitted by least-squares linear regression using $1/X^2$ as a weighting factor. The lower limit of quantification (LLOQ) was defined as the lowest concentration on the calibration curve with a RSD less than 20% and a deviation from the nominal concentration within ± 20%. The LLOQ was determined in six replicates. The stability of the berberine and its nine metabolites in plasma samples was evaluated by analyzing triplicates of QC samples at low and high concentration levels stored at room temperature for 4 h (bench-top stability), at −80°C for 21 days (long-term stability), after undergoing three freeze–thaw cycles (freeze and thaw stability), and post preparative QC samples stored at 10°C (autosampler temperature) for 12 h (post-preparative stability), respectively.

Table S1. Regression equation, linear range and LLOQ for LC-MS/MS analysis of berberine and its nine metabolites in rat plasma.

Analytes	Regression equation	*r*	Linear range (ng/mL)	LLOQ		
				Nominal concentration (ng/mL)	RSD (%)	RE (%)
Berberine	y=0.0237x+0.00771	0.9995	0.500-100	0.500	8.4	-4.2
M1	y=0.0522x+0.000695	0.9995	0.500-100	0.500	3.1	2.0
M2	y=0.00978x+0.000216	0.9994	0.500-100	0.500	5.5	4.0
M3	y=0.0369x+0.000324	0.9998	0.500-100	0.500	4.0	7.0
M4	y=0.0144x+0.000258	0.9992	1.00-500	1.00	7.0	2.0
M5	y=0.0284x+0.00112	0.9981	1.00-500	1.00	4.0	0.0
M6	y=0.0101x+0.000268	0.9995	1.00-500	1.00	4.9	3.0
M7	y=0.00354x+0.00042	0.9984	1.00-500	1.00	8.7	-0.6
M8	y=0.00181x+0.000377	0.9994	1.00-500	1.00	10.3	-0.5
M9	y=0.0122x+0.000802	0.9991	1.00-500	1.00	5.5	2.0

Table S2. Precision, accuracy, extraction recovery and matrix effect for LC-MS/MS analysis of berberine and its nine metabolites in rat plasma.

Aanalytes	Nominal concentration (ng/mL)	Precision		Accuracy	Extraction recovery		Matrix effect	
		Intra-day RSD (%)	Inter-day RSD (%)	RE (%)	Mean (%)	RSD (%)	Mean (%)	RSD (%)
Berberine	1.50	3.6	9.4	-4.3	71.4	3.9	109	4.6
	8.00	3.4	3.0	3.5	71.8	3.8	-	-
	80.0	1.8	2.6	3.1	76.0	11.5	102	1.6
M1	1.50	4.9	4.7	6.0	70.8	3.3	105	1.5
	8.00	1.1	2.0	4.0	72.4	3.6	-	-
	80.0	2.4	2.0	3.9	69.8	2.3	103	1.6
M2	1.50	2.8	2.2	-4.9	70.2	2.5	107	2.2
	8.00	2.0	2.4	6.3	72.7	1.8	-	-
	80.0	1.4	1.8	5.5	71.5	1.5	103	0.8
M3	1.50	2.4	2.7	6.7	71.3	3.0	104	2.4
	8.00	1.4	1.2	5.2	72.5	0.7	-	-
	80.0	1.2	1.7	2.8	69.8	0.7	102	1.1
M4	3.00	5.7	8.6	-11.4	79.3	8.4	104	0.7
	40.0	2.0	3.0	0.2	80.8	2.7	-	-
	400	1.5	1.5	9.8	86.4	12.4	92.5	1.5
M5	3.00	4.9	7.2	-10.3	82.3	9.2	97.4	2.9
	40.0	2.2	2.0	3.5	81.0	2.0	-	-
	400	1.5	1.4	13.8	94.7	7.8	93.5	3.1
M6	3.00	7.2	7.6	-9.8	76.8	8.7	101	1.5
	40.0	2.3	2.0	0.2	80.2	2.5	-	-
	400	1.5	1.7	6.8	85.1	11.2	93.0	1.4
M7	3.00	3.9	8.0	-9.8	77.6	5.1	103	2.7
	40.0	2.4	2.5	-1.3	81.2	1.8	-	-
	400	1.6	2.0	9.5	86.8	12.8	97.2	2.4
M8	3.00	7.7	8.0	-9.5	78.4	2.8	109	3.1
	40.0	3.0	2.4	1.2	82.1	2.9	-	-
	400	1.9	3.2	3.5	91.7	14.3	95.7	1.6
M9	3.00	6.0	7.6	-10.3	77.6	9.2	104	1.6

	40.0	2.6	2.0	-0.8	80.5	1.4	-	-
	400	1.4	1.5	7.2	84.8	11.2	93.0	1.9

Table S3. Stability of berberine and its nine metabolites in rat plasma.

Analytes	Nominal concentration (ng/mL)	Post-preparative stability		Bench-top stability		Freeze-thaw stability		Long-term stability	
		RE (%)	RSD (%)	RE (%)	RSD (%)	RE (%)	RSD (%)	RE (%)	RSD (%)
Berberine	1.50	2.7	1.9	-2.0	4.8	7.3	4.5	3.3	2.7
	80.0	7.6	2.4	2.0	4.6	8.6	2.5	6.4	2.5
M1	1.50	1.3	3.8	-0.7	2.0	-0.7	2.5	0.0	0.8
	80.0	7.9	1.3	6.9	3.6	8.0	0.5	5.5	1.5
M2	1.50	-13.3	3.2	-12.7	3.0	-11.3	2.3	-13.3	2.2
	80.0	10.9	1.2	6.0	2.0	9.8	3.0	8.8	1.9
M3	1.50	9.3	1.7	1.3	4.0	7.3	0.9	6.7	1.9
	80.0	7.1	1.2	0.9	2.8	7.9	4.9	4.6	2.0
M4	3.00	-2.0	2.1	-3.0	0.5	-2.7	5.7	1.7	2.2
	400	9.2	2.4	8.0	1.4	2.0	2.3	6.5	2.9
M5	3.00	-4.0	1.7	-3.7	2.1	-1.3	5.4	3.0	2.9
	400	13.5	0.8	13.0	2.2	12.0	0.6	13.0	1.6
M6	3.00	-2.3	3.7	-2.3	1.7	1.7	3.9	3.7	1.4
	400	7.5	2.4	7.0	1.3	5.5	1.9	6.2	2.5
M7	3.00	-2.0	3.0	-2.0	2.0	5.0	3.9	3.0	3.6
	400	7.2	2.6	6.8	1.6	4.2	2.2	6.2	2.5
M8	3.00	-1.0	5.7	-1.7	8.1	-2.3	8.5	-1.7	6.5
	400	4.8	2.0	7.8	1.7	4.0	2.5	8.2	3.0
M9	3.00	-2.3	2.3	-2.3	2.8	-1.3	2.2	3.7	2.6
	400	8.2	2.0	5.8	0.4	6.2	2.7	6.8	1.9

Table S4. Dilution effect on the quantification of high concentration plasma samples.

Analytes	Spiked *C.* (ng/mL)	Dilution factor	Measured *C.* (ng/mL)	RSD (%)	Accuracy (%)
Berberine	1350	15	91.9 ± 7.05	7.7	2.2
	750	15	49.8 ± 3.21	6.4	-0.4
	300	15	21.4 ± 2.02	9.4	7.0
M7	900	2	458 ± 34.1	7.4	1.8
	700	2	337 ± 28.4	8.4	-3.6
	500	2	260 ± 13.0	5.0	4.0

Table S5. The excretion results of berberine and its nine metabolites in rat urine (Mean ± SD, *n* = 6).

Time (h)	Berberine		M1		M2		M3		M4	
	Excretion Amounts (ng)	Accumulative Excretion (% of dose)	Excretion Amounts (ng)	Accumulative Excretion (% of dose)	Excretion Amounts (ng)	Accumulative Excretion (% of dose)	Excretion Amounts (ng)	Accumulative Excretion (% of dose)	Excretion Amounts (ng)	Accumulative Excretion (% of dose)
0-4	21.4 ± 19.5	0.000219 ± 0.000209	2280 ± 1188	0.0246 ± 0.0139	1931 ± 1562	0.0200 ± 0.0155	14.7 ± 4.09	0.000148 ± 0.0000338	2131 ± 2034	0.0140 ± 0.0129
4-8	30.5 ± 16.9	0.000312 ± 0.000179	6427 ± 2886	0.0690 ± 0.0307	10240 ± 6426	0.110 ± 0.0737	35.4 ± 29.8	0.000456 ± 0.000295	5323 ± 4379	0.0355 ± 0.0292
8-12	79.6 ± 49.6	0.00081 ± 0.000536	11822 ± 2482	0.135 ± 0.0106	10431 ± 6619	0.121 ± 0.0744	83.9 ± 28.1	0.000914 ± 0.000225	10313 ± 3402	0.0677 ± 0.0229
12-24	249 ± 184	0.00252 ± 0.00197	38955 ± 15019	0.399 ± 0.1389	26275 ± 14065	0.268 ± 0.137	163 ± 69.4	0.00160 ± 0.000652	14925 ± 3431	0.0969 ± 0.0211
24-36	339±215	0.00342 ± 0.00224	50190 ± 17454	0.516 ± 0.1618	30026 ± 14048	0.307 ± 0.140	189 ± 80.3	0.00186 ± 0.000767	16221 ± 3739	0.106 ± 0.0246
36-48	743±545	0.00748 ± 0.00546	112239 ± 50265	1.17 ± 0.537	36394 ± 17115	0.374 ± 0.178	278 ± 82.5	0.00224 ± 0.000775	19533 ± 5687	0.128 ± 0.0408
48-60	833±590	0.00836 ± 0.00587	116146 ± 52583	1.21 ± 0.567	37499 ± 17090	0.386 ± 0.178	269 ± 98.1	0.00264 ± 0.000906	22052 ± 4429	0.144 ± 0.0326
60-72	904±599	0.00907 ± 0.00598	118246 ± 52055	1.23 ± 0.561	38517 ± 17503	0.397 ± 0.184	283 ± 105	0.00279 ± 0.000986	23176 ± 4231	0.151 ± 0.0312
72-84	1006±530	0.0101 ± 0.00526	119586 ± 53091	1.24 ± 0.574	38719 ± 17509	0.399 ± 0.184	288 ± 109	0.00284 ± 0.00102	23432 ± 4283	0.153 ± 0.0316

Time (h)	M5		M6		M7		M8		M9	
	Excretion Amounts (ng)	Accumulative Excretion (% of dose)	Excretion Amounts (ng)	Accumulative Excretion (% of dose)	Excretion Amounts (ng)	Accumulative Excretion (% of dose)	Excretion Amounts (ng)	Accumulative Excretion (% of dose)	Excretion Amounts (ng)	Accumulative Excretion (% of dose)
0-4	4.14 ± 0.838	0.0000338 ± 0.00000510	3202 ± 284	0.0219 ± 0.0178	12284 ± 7744	0.0877 ± 0.0553	2190 ± 794	0.0188 ± 0.0077	2604 ± 1338	0.0180 ± 0.00990
4-8	4.93 ± 4.01	0.0000401 ± 0.0000314	6508 ± 5525	0.0446 ± 0.0367	26102 ± 14551	0.181 ± 0.107	3407 ± 1996	0.0288 ± 0.0156	6988 ± 6292	0.0520 ± 0.0549
8-12	8.08 ± 4.47	0.0000654 ± 0.0000356	12810 ± 5495	0.0869 ± 0.0368	75466 ± 16076	0.514 ± 0.125	4336 ± 2860	0.0365 ± 0.0240	16481 ± 7640	0.111 ± 0.0522
12-24	12.6 ± 7.03	0.000101 ± 0.0000535	16971 ± 3253	0.114 ± 0.0227	264775 ± 99146	1.78 ± 0.621	6872 ± 3236	0.0571 ± 0.0272	20379 ± 9081	0.137 ± 0.0619
24-36	14.8 ± 6.76	0.000119 ± 0.000052	18694 ± 3212	0.126 ± 0.0251	350745 ± 100901	2.36 ± 0.605	7719 ± 3355	0.0643 ± 0.0282	22415 ± 10763	0.151 ± 0.0743
36-48	26.1 ± 11.0	0.000210 ± 0.0000932	21146 ± 4154	0.143 ± 0.0332	431459 ± 94825	2.93 ± 0.679	11221 ± 4194	0.0943 ± 0.0385	23505 ± 10694	0.159 ± 0.0745
48-60	27.9 ± 10.4	0.000225 ± 0.0000892	23909 ± 3835	0.162 ± 0.0315	532181 ± 78480	3.61 ± 0.545	11624 ± 4168	0.0976 ± 0.0385	28746 ± 7998	0.194 ± 0.055
60-72	29.7 ± 9.33	0.000239 ± 0.0000817	24761 ± 3994	0.167 ± 0.0329	594065 ± 68748	4.03 ± 0.507	12076 ± 4157	0.101 ± 0.0387	30008 ± 7116	0.202 ± 0.0495
72-84	30.4 ± 9.39	0.000245 ± 0.0000826	24962 ± 4085	0.169 ± 0.0336	621133 ± 55793	4.21 ± 0.463	12177 ± 4205	0.102 ± 0.0392	30390 ± 7091	0.204 ± 0.0496

Table S6. The excretion results of berberine and its nine metabolites in rat bile (Mean ± SD, *n* = 6).

Time (h)	Berberine		M1		M2		M3		M4	
	Excretion Amounts (ng)	Accumulative Excretion (% of dose)	Excretion Amounts (ng)	Accumulative Excretion (% of dose)	Excretion Amounts (ng)	Accumulative Excretion (% of dose)	Excretion Amounts (ng)	Accumulative Excretion (% of dose)	Excretion Amounts (ng)	Accumulative Excretion (% of dose)
0-2	2281 ± 1256	0.0227 ± 0.0120	11408 ± 3818	0.119 ± 0.037	168 ± 42.3	0.00174 ± 0.000460	290 ± 114	0.00287 ± 0.00105	3250 ± 1411	0.0212 ± 0.0087
2-4	4903 ± 2118	0.0488 ± 0.0199	29658 ± 3594	0.311 ± 0.026	366 ± 115	0.00382 ± 0.00116	842 ± 211	0.00837 ± 0.00181	8745 ± 2527	0.0572 ± 0.0148
4-8	9117 ± 3847	0.0908 ± 0.0359	67737 ± 8416	0.711 ± 0.078	840 ± 256	0.00878 ± 0.00264	1910 ± 512	0.0190 ± 0.00451	19081 ± 6374	0.125 ± 0.0385
8-12	11628 ± 4925	0.116 ± 0.046	99189 ± 15401	1.04 ± 0.163	1213 ± 356	0.0127 ± 0.00384	2735 ± 723	0.0272 ± 0.00643	26730 ± 9322	0.175 ± 0.0569
12-24	15763 ± 8352	0.157 ± 0.0794	191377 ± 17706	2.01 ± 0.217	2456 ± 581	0.0258 ± 0.00650	5008 ± 1291	0.0499 ± 0.0115	54170 ± 21355	0.355 ± 0.132
24-36	17505 ± 9244	0.174 ± 0.0878	248827 ± 30993	2.62 ± 0.371	3237 ± 526	0.0339 ± 0.00652	6252 ± 1401	0.0623 ± 0.0121	71630 ± 34912	0.470 ± 0.219

Time (h)	M5		M6		M7		M8		M9	
	Excretion Amounts (ng)	Accumulative Excretion (% of dose)	Excretion Amounts (ng)	Accumulative Excretion (% of dose)	Excretion Amounts (ng)	Accumulative Excretion (% of dose)	Excretion Amounts (ng)	Accumulative Excretion (% of dose)	Excretion Amounts (ng)	Accumulative Excretion (% of dose)
0-2	78.2 ± 32.9	0.000628 ± 0.000255	3490 ± 1827	0.0248 ± 0.0142	10397 ± 3342	0.0744 ± 0.0301	8096 ± 3128	0.0674 ± 0.0248	4357 ± 2219	0.0294 ± 0.0144
2-4	234 ± 99.2	0.00189 ± 0.000759	11949 ± 3343	0.0844 ± 0.0261	17154 ± 4655	0.1227 ± 0.0437	18724 ± 7350	0.156 ± 0.0589	8221 ± 3220	0.0557 ± 0.0212
4-8	578 ± 276	0.00465 ± 0.00211	27793 ± 8234	0.197 ± 0.066	25250 ± 7087	0.1805 ± 0.0653	38691 ± 14718	0.323 ± 0.120	14489 ± 5547	0.0982 ± 0.0375
8-12	882 ± 375	0.00709 ± 0.00287	37950 ± 13211	0.270 ± 0.111	30242 ± 7929	0.216 ± 0.0743	55727 ± 19508	0.466 ± 0.162	19799 ± 7270	0.134 ± 0.0498
12-24	2067 ± 667	0.0167 ± 0.00505	49773 ± 7909	0.353 ± 0.0747	43398 ± 10830	0.310 ± 0.104	120839 ± 28139	1.02 ± 0.249	42379 ± 12863	0.288 ± 0.0881
24-36	2465 ± 712	0.0200 ± 0.00573	56648 ± 9477	0.401 ± 0.0826	51135 ± 12145	0.365 ± 0.118	161777 ± 25193	1.36 ± 0.242	63655 ± 18241	0.430 ± 0.118

Table S7. The excretion results of berberine and its nine metabolites in rat feces (Mean ± SD, *n* = 6).

Time (h)	Berberine		M1		M2		M3	
	Excretion Amounts (ng)	Accumulative Excretion (% of dose)	Excretion Amounts (ng)	Accumulative Excretion (% of dose)	Excretion Amounts (ng)	Accumulative Excretion (% of dose)	Excretion Amounts (ng)	Accumulative Excretion (% of dose)
0-84	841573 ± 119494	8.43 ± 1.10	1494340 ± 788797	18.6 ± 4.01	141242 ± 77630	1.49 ± 0.819	21284 ± 10492	0.254 ± 0.055

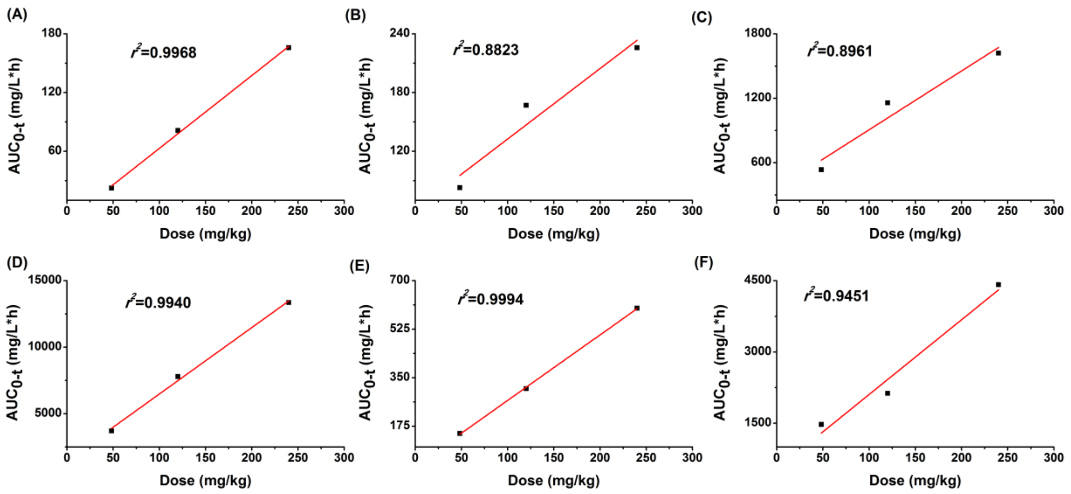

Figure S1. Linear regressions showing dose proportionality in terms of dose and AUCs for berberine (A), M1 (B), M6 (C), M7 (D), M8 (E) and M9 (F).

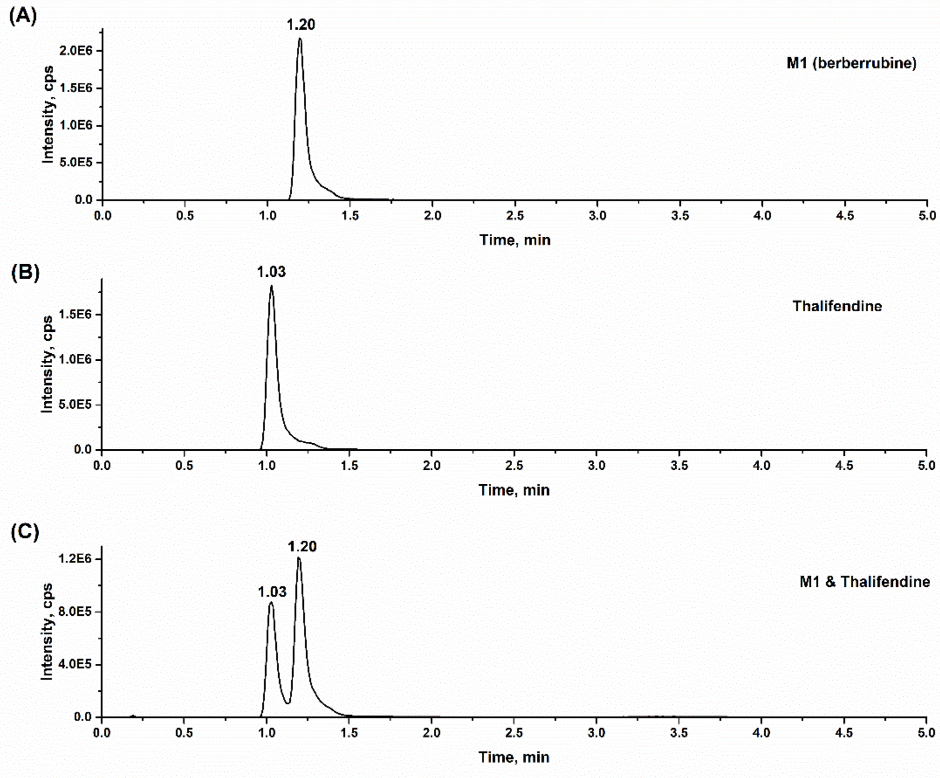

Figure S2. Typical MRM chromatograms of M1 (berberrubine) and thalifendine standard. (A) M1 standard; (B) thalifendine standard; (C) mixture standard of M1 and thalifendine.
